# Radiobiological Comparison of Teardrop Technique for Breast Cancer Radiotherapy Treatment Planning on a Tomotherapy System

**DOI:** 10.7759/cureus.14390

**Published:** 2021-04-09

**Authors:** Higmar Herrera, Uvaldo Reyes

**Affiliations:** 1 Radiation Oncology, Centro Estatal De Cancerología De Durango, Victoria de Durango, MEX

**Keywords:** breast cancer, treatment planning, radiobiological parameters, utcp, geud

## Abstract

Breast cancer is one of the most common cancer worldwide with large morbidity. In Mexico, it is the first cause of death by cancer in women. Radiotherapy has proven to be a great tool to control such ailments and TomoTherapy is a relatively new technology to accomplish it. To obtain good clinical outcomes, tight dosimetric constraints are placed on organs at risk (OARs) to maximize tumor control and minimize normal tissue complication probabilities. The teardrop technique helps meeting these constraints by placing a virtual block over parts of the ipsilateral lung and the heart but it contributes to lengthen the treatment time. In this work, we present our experience in using this technique and compare its radiobiological estimations with similar plans without it. Ten patients diagnosed with breast cancer were planned twice, with and without the teardrop technique. Dose-volume histograms were obtained and analyzed to get uncomplicated tumor control probability (UTCP) and optimization estimator (fEUD) parameters. Classical dosimetrical parameters for planning target volumes (PTVs): conformity index, homogeneity index, and coverage were also recorded and statistically described. Several dosimetrical parameters for OARs were recorded and analyzed. The UTCP parameter had a mean value of 0.968 ± 0.023 when no block was used and 0.966 ± 0.022 with the teardrop. The fEUD parameter values were: 0.515 ± 0.049 without blocks and 0.541 ± 0.057 with the teardrop. Optimization of every plan was stopped only after all constraints were met, and it was easier to accomplish this goal with the teardrop technique. The teardrop technique permitted a 5% gain in fEUD. The teardrop technique was observed to have a net radiobiological benefit with little impact on patient scheduling.

## Introduction

Breast cancer is one of the most common cancer worldwide with large morbidity. In Mexico, it is the first cause of death by cancer in women. Radiotherapy has proven to be a great tool to control such ailment and TomoTherapy is a relatively new technology to accomplish it. To obtain good clinical outcomes, tight dosimetric constraints are used in the optimization algorithm for organs at risk (OARs) to maximize tumor control and minimize normal tissue complication probabilities. The teardrop technique as described by Matson [1] consists of placing a virtual block over parts of the ipsilateral lung and the heart. This block resembles an inverted teardrop that extends from about 2.5 cm of the first slice where the ipsilateral lung appears to the last slice where the PTV ends. The thinner part being a rounded margin of the spine and the thicker one fanning from the most internal edge of the PTV to its most external edge in every slice at about 2.5 cm from the proximal PTV edge. The teardrop is defined as a complete block, meaning that no beamlet can traverse it. It helps meeting the imposed constraints but it contributes to lengthen the treatment time. In this work, we present our experience in using this technique and compare its radiobiological estimations with similar plans without it [2].

## Materials and methods

To keep our treatment times as short as possible we introduced some modifications to the technique: (i) always use a 5 cm field width and (ii) reduce the extension of the teardrop at least 5 cm from the last slice where the infraclavicular nodes appear.

Ten patients diagnosed with breast cancer were planned twice over basal CT images with no breath-hold, with and without the teardrop technique. Five patients were prescribed 50 Gy in 25 fractions, three left breasts and two right. Four patients were prescribed 40 Gy in 15 fractions, three left and one right. One patient was prescribed 40 Gy in 15 fractions with an integrated boost of 45 Gy in 15 fractions to the right breast. Dose-volume histograms were obtained on Accuray Planning Station (Version 5.1.1.6, Accuray, Sunnyvale, CA) for TomoHDA (Version 2.1.2, Accuray, Palo Alto, CA) and analyzed on Albireo Target Cygnus X1 (Version 4.0.2008, Carlos Haya Regional University Hospital, Malaga, Spain) to get uncomplicated tumor control probability (UTCP) and an optimization estimator based on generalized equivalent uniform dose (fEUD) parameter [3-5]. This estimator is defined as:

$fEUD = \frac{1}{\left ( 1+\frac{EUD_{0}}{EUD_{tumor}} \right )^{n}}\prod \frac{1}{\left ( 1+\frac{EUD_{OAR}}{EUD_{OAR0}} \right )^{n}}$

where EUD_0_ is the prescribed dose, EUD_OAR0_ is the maximum uniform tolerable dose for the OAR and n is an importance factor.

Matlab Statistics Toolbox (Version 6.0.0.88 release 12, MathWorks, Inc., Natick, MA) was used to examine the significance of the results through bootstrapping of Student’s and Wilcoxson’s tests. OAR considered were the heart, contralateral breast, and both (total) lungs [6]. The same statistical tests were applied to the treatment delivery time. Classical dosimetric parameters for planning target volumes (PTVs): conformity index, homogeneity index, and coverage were also recorded and statistically described. Several dosimetric parameters for OARs listed in Table 1 were recorded and analyzed.

**Table 1:** Recorded dosimetric parameters for OARs. OARs: organs at risk, D_mean_: mean dose, V_x_: volume (%) getting x Gy or more.

OARs	Parameters
Heart	D_mean _(Gy), V_5_, V_20_, V_25_, V_35 _(%)
Contralateral breast	D_mean _(Gy), V_5_, V_10_, V_15 _(%)
Total lungs	D_mean _(Gy), V_5_, V_20 _(%)

## Results

**Figure 1:**
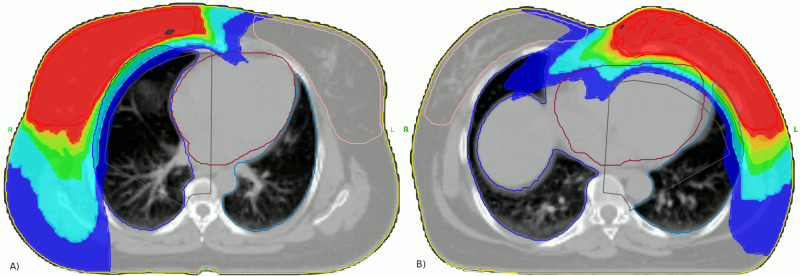
Typical dose distributions obtained with the teardrop technique. (A) Image depicting a typical dose distribution for a left breast cancer patient; (B) image depicting a typical dose distribution for a right breast cancer patient.

Typical dose distribution for right and left breasts are shown in Figure 1.

UTCP for the unblocked plans ranged from 0.919 to 0.989, while the same parameter for teardrop plans ranged from 0.920 to 0.989. fEUD ranged from 0.480 to 0.648 and 0.479 to 0.672. The average values for all radiobiological and PTVs dosimetric parameters are displayed in Table 2.

**Table 2:** Average values and standard deviations observed on the radiobiological and dosimetric parameters for PTVs PTVs: planning target volumes, UTCP: uncomplicated tumor control probability, fEUD: radiobiological optimization parameter based on generalized equivalent uniform dose.

Parameter	No block	Teardrop
UTCP	0.968 ± 0.023	0.966 ± 0.022
fEUD	0.515 ± 0.049	0.541 ± 0.057
Conformity index	1.39 ± 0.16	1.46 ± 0.15
Homogeneity index	0.076 ± 0.004	0.077 ± 0.007
Coverage (%)	95.3 ± 0.5	96.2 ± 0.8
Treatment time (s)	361.0 ± 75.3	494.3 ± 68.5

**Figure 2:**
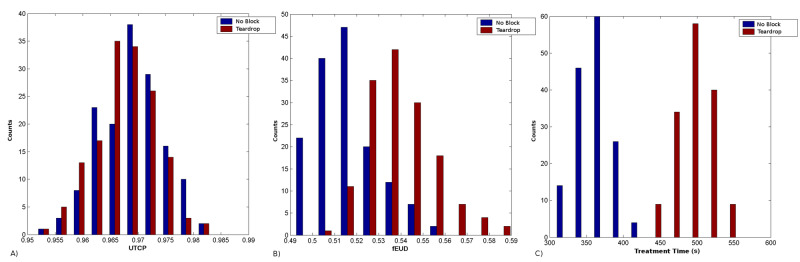
Histograms from 150 bootstrapping samples for: (A) radiobiological parameter UTCP, (B) radiobiological parameter fEUD, and (C) treatment time. (A) Histogram of a 150 sampling of UTCP radiobiological parameter; (B) histogram of a 150 sampling of fEUD radiobiological parameter; (C) histogram of a 150 sampling of treatment time. UTCP: uncomplicated tumor control probability, fEUD: radiobiological optimization parameter based on generalized equivalent uniform dose.

The bootstrap results for radiobiological parameters and treatment time are shown in Figure 2. The observed differences were significant with 95% confidence (pfEUDStudent=0.004, pfEUDWilcoxon=0.009; pTimeStudent=0.002, pTimeWilcoxon=0.011).

The dosimetric parameter values for the OARs are listed in Table 3.

**Table 3:** Average values and standard deviations of the recorded dosimetric parameters for OARs. OARs: organs at risk, D_mean_: mean dose, V_**_: volumes related to the dose-volume histogram.

Heart			Contralateral breast			Total lungs		
	No block	Teardrop		No block	Teardrop		No block	Teardrop
D_mean_	8.0 ± 1.8	5.5 ± 1.7	D_mean_	7.9 ± 1.4	6.8 ± 0.8	D_mean_	10.0 ± 0.8	8.8 ± 0.9
V_5_	67.5 ± 22.5	35.4 ± 15.5	V_5_	77.7 ± 17.1	61.9 ± 18.0	V_5_	64.1 ± 4.9	54.7 ± 7.6
V_20_	4.9 ± 4.6	3.2 ± 3.4	V_10_	22.2 ± 9.8	15.2 ± 4.5	V_20_	12.6 ± 2.0	11.0 ± 2.3
V_25_	2.6 ± 3.1	1.6 ± 2.1	V_15_	7.0 ± 4.6	4.9 ± 3.0			
V_35_	0.5 ± 1.0	0.3 ± 0.6						

## Discussion

Optimization of every plan was stopped only after all constraints were met, and it was easier to accomplish this goal with the teardrop technique. It was not possible to accurately measure treatment planning completion time but the number of iterations required to obtain the final plan was reduced by at least 1000 iterations when using the teardrop. No statistically significant difference was observed for the UTCP, but the teardrop technique resulted in a 5% gain in fEUD. However, this gain came with a 37% increase in treatment delivery time which is still below the internal standard of 600 seconds allocation time established for our patients. The treatment times obtained are below those reported by Matson and it may be related to our usage of 5 cm field width and a different overlap priority ordering.

As can be seen in Table 3, all dosimetric parameters registered for OARs improved when using the teardrop technique even for the contralateral breast. We think that improved sparing of the contralateral breast is due to the fact that as the block does not allow irradiation from any beamlet that traverses this structure before and after reaching the PTV as from the beam eye view, it effectively blocks part of the breast that would be irradiated without the teardrop.

## Conclusions

We have presented our initial experience on the use of the teardrop technique and have observed it to have a net radiobiological benefit with little impact on patient scheduling by maintaining treatment times under 600 seconds. This has allowed us to reduce the treatment planning time and improve the quality of the treatments we offer to breast cancer patients.
